# Glucagon Potentiates Insulin Secretion Via β-Cell GCGR at Physiological Concentrations of Glucose

**DOI:** 10.3390/cells10092495

**Published:** 2021-09-21

**Authors:** Yulin Zhang, Chengsheng Han, Wenzhen Zhu, Guoyi Yang, Xiaohong Peng, Sohum Mehta, Jin Zhang, Liangyi Chen, Yanmei Liu

**Affiliations:** 1 State Key Laboratory of Membrane Biology, Beijing Key Laboratory of Cardiometabolic Molecular Medicine, Institute of Molecular Medicine, College of Future Technology, Peking University, Beijing 100871, China; 1906390239@pku.edu.cn (Y.Z.); hanchengsheng@pku.edu.cn (C.H.); 1201110443@pku.edu.cn (W.Z.); gyyang0611@pku.edu.cn (G.Y.); 2006388450@pku.edu.cn (X.P.); 2 Department of Pharmacology, University of California San Diego, La Jolla, CA 92093-0702, USA; sohum@ucsd.edu (S.M.); jzhang32@ucsd.edu (J.Z.); 3 PKU-IDG/McGovern Institute for Brain Research, Beijing 100871, China; 4 Beijing Academy of Artificial Intelligence, Beijing 100871, China; 5 Key Laboratory of Brain, Cognition and Education Sciences, Ministry of Education, Institute for Brain Research and Rehabilitation, South China Normal University, Guangzhou 510631, China

**Keywords:** glucagon, GCGR, GLP-1R, α-cells, β-cells, glucose-stimulated insulin secretion, cAMP

## Abstract

Incretin-potentiated glucose-stimulated insulin secretion (GSIS) is critical to maintaining euglycemia, of which GLP-1 receptor (GLP-1R) on β-cells plays an indispensable role. Recently, α-cell-derived glucagon but not intestine-derived GLP-1 has been proposed as the critical hormone that potentiates GSIS via GLP-1R. However, the function of glucagon receptors (GCGR) on β-cells remains elusive. Here, using GCGR or GLP-1R antagonists, in combination with glucagon, to treat single β-cells, α-β cell clusters and isolated islets, we found that glucagon potentiates insulin secretion via β-cell GCGR at physiological but not high concentrations of glucose. Furthermore, we transfected primary mouse β-cells with RAB-ICUE (a genetically encoded cAMP fluorescence indicator) to monitor cAMP level after glucose stimulation and GCGR activation. Using specific inhibitors of different adenylyl cyclase (AC) family members, we revealed that high glucose concentration or GCGR activation independently evoked cAMP elevation via AC5 in β-cells, thus high glucose stimulation bypassed GCGR in promoting insulin secretion. Additionally, we generated β-cell-specific GCGR knockout mice which glucose intolerance was more severe when fed a high-fat diet (HFD). We further found that β-cell GCGR activation promoted GSIS more than GLP-1R in HFD, indicating the critical role of GCGR in maintaining glucose homeostasis during nutrient overload.

## 1. Introduction

Insulin released from pancreatic β-cells, which facilitates glucose uptake and utilization in downstream tissues, such as liver, adipocytes, and muscle, is critical in maintaining tight glucose homeostasis [1]. Orally induced blood glucose elevation triggers two- to three-fold more insulin secretion than intravenous glucose injection [2], which is ascribed to the stimulatory effects of intestine-derived incretins, such as glucose-dependent insulinotropic peptide (GIP) and glucagon-like peptide-1 (GLP-1) [3,4]. In humans, GLP-1 is more potent than GIP at stimulating insulin release [5], and its stimulatory effects persist in type 2 diabetes (T2D) patients, while those of GIP diminish [6]. Thus, GLP-1 is widely accepted as an essential incretin for blood glucose homeostasis, and its receptor (GLP-1R) serves as a therapeutic drug target for T2D.

Recently, the critical function of intestine-derived GLP-1 has been challenged. Pancreatic β-cell-specific GLP-1R knockout mice showed normal oral glucose tolerance but impaired intraperitoneal glucose tolerance, indicating an indispensable role of islet-derived rather than intestine-derived ligands in activating GLP-1R in β-cells during glucose stimulation [7]. 

GLP-1 is found in pancreatic α-cells [8,9,10,11], colocalizing with glucagon in the granules of murine α-cells [12]. Thus α-cell-derived GLP-1 is proposed to potently enhance glucose-stimulated insulin secretion (GSIS) [7,13,14]. However, the relative concentrations of GLP-1 and glucagon in α-cells vary in different studies [10,11,15], and how α-cells preferentially release GLP-1 or glucagon from the same granule is also obscure. Glucagon, on the other hand, also stimulates insulin secretion [16] via generating cAMP by binding to both the glucagon receptor (GCGR) and GLP-1R [17,18,19,20] on β-cells. Interestingly, recent studies show that the β-cell GCGR knockout mice exhibit normoglycaemia [18,19,20]. Glucagon enhanced GSIS in 10 mM [19] or 12 mM [18] glucose-induced GCGR knockout islets was not different from control islets, suggesting that the major insulinotropic effect of glucagon is achieved via GLP-1R. However, as the cognate downstream receptor of glucagon, the physiological significance of β-cell GCGR remains unclear, and how it functions under metabolic stress has yet to be established.

This study shows that besides GLP-1R, GCGR also contributes to glucagon-enhanced GSIS at physiological glucose concentrations. However, at high glucose levels, glucagon-enhanced GSIS results from GLP-1R only. We found that the possible mechanism may be that high glucose and GCGR activate the same pool of adenylyl cyclase (AC) AC5, increasing cAMP to enhance insulin secretion, while GLP-1R seems to recruit other ACs. By generating and characterizing β-cell-specific GCGR knockout mice, we confirmed GCGR’s effect on insulin secretion at physiological concentrations of glucose. We further found that downstream of glucagon, β-cell GCGR plays a more prevalent role than GLP-1R in potentiating GSIS in mice fed the high-fat diet (HFD). These data highlight the essential roles of β-cell GCGR in mediating both glucose homeostasis and metabolic status. 

## 2. Materials and Methods

### 2.1. Animals

GluCre-ROSA26EYFP mice were a kindly gifted by Prof. Herbert Y. Gaisano from the University of Toronto (Toronto, Ontario, Canada). For the generation of *Gcgr^f/f^* mice, the targeting vector was constructed by inserting an Frt-flanked neomycin cassette in the third intron of *Gcgr* and two loxP sites in the locations described in Figure 3A, then electroporated into embryonic stem cells from C57BL/6J mice (Beijing Biocytogen Co., Ltd, Beijing, China). The founder mice were mated with an flp-deleter mouse (Jackson Laboratory; stock number 003946) to remove the neomycin cassette. β-cell-specific GCGR knockout (*Gcgr^βcell−/−^*) mice were generated by crossbreeding *Gcgr^f/f^* mice with ins1-cre mice (Jackson Laboratory; stock number 026801) [21]. Upon loxP recombination, exons 4 to 14 of the *Gcgr* gene were removed in β cells. Mice were maintained in one cage with a light/dark cycle of 12 h and administered a chow diet, ad libitum. Only male mice older than 8 weeks were used in our study and were fed either a normal diet (ND) or HFD with 60% kcal fat (D12492, Research Diets) for 3 months before the experiments. 

### 2.2. IPGTT

Blood samples were collected via the tail nick for the intraperitoneal glucose tolerance test (IPGTT), and blood glucose was measured with a blood glucose meter (ACCU-CHEK^®^ Active, Roche, Basel, Switzerland). Mice were fasted for 18 h to achieve relatively low basal glucose levels. Then glucose (G7021, Sigma, Shanghai, China) was administered by intraperitoneal (IP) injection at different doses (1 g/kg, 2 g/kg, and 4 g/kg). Glycaemia was measured at 15, 30, 45, 60, and 120 min after glucose administration.

### 2.3. Isolation of Mouse Islets and Dissociation of the Islets into Individual β-Cells or Cell Clusters

Pancreatic islets were isolated from mice as previously described [22]. After overnight culture in RPMI 1640 medium (10% FBS, C22400500BT, Gibco), islets were washed with Hank’s balanced salt solution (HBSS) without calcium and magnesium (5.3 mM KCl, 0.44 mM KH_2_PO_4_, 4.16 mM NaHCO_3_, 137.9 mM NaCl, 0.34 mM Na_2_HPO_4_ and 5 mM glucose, pH 7.4) and then treated with 0.25% trypsin (25200-056, Life Technologies) for 3 min at 37 °C. The cells were plated on coverslips coated with poly-L-lysine (P4707, Sigma) and cultured in RPMI 1640 medium (10% FBS, C22400500BT, Gibco) at 37 °C and 5% CO_2_ for 24–48 h before experiments.

### 2.4. Detection of Hormone Secretion from Islets

After culturing overnight, five similar-sized islets were selected and randomly assigned to each group. After preincubation in KRBB (125 mM NaCl, 5.9 mM KCl, 2.56 mM CaCl_2_, 1.2 mM MgCl_2_, 1 mM L-glutamine, 25 mM HEPES, 1 g/L BSA, 3 mM glucose) for 1 h at 37 °C, the islets were transferred to another 200 µL of KRBB supplemented with the indicated glucose concentrations (3 mM, 5 mM, 7 mM, 11 mM and 20 mM) for 1 h at 37 °C. Additionally, 1 µM MK0893 [23] or 1 µM Exendin 9-39 [18,24,25] was included to block GCGR or GLP-1R that was activated by 100 nM glucagon. The incubation solution was then collected, and insulin levels were measured using a rat/mouse insulin ELISA kit according to the manufacturer’s instructions (EZRMI-13K, Millipore). Insulin secretion was normalized to ng/islet/h. The key reagent information is as followed (Table 1).

For glucagon secretion measurements, 25 islets were incubated for 4 h in 200 µL of KRBB containing 7 mM glucose. The glucagon level was measured using a glucagon ELISA kit (10-1271-01, Mercodia, Uppsala, Sweden).

### 2.5. Electrophysiology and Membrane Capacitance Recording

Perforated whole-cell recordings of membrane capacitance (Cm) were used to assess the secretion abilities of single β-cells [26,27]. β-cells with a Cm above 4 pF were selected for experiments. The standard extracellular solution contained 118 mM NaCl, 20 mM tetraethylammonium chloride (TEA), 5.6 mM KCl, 2.6 mM CaCl_2_·2H_2_O, 1.2 mM MgCl_2_, 5- or 20-mM D-glucose and 5 mM HEPES at pH 7.4. The intracellular solution contained 152 mM CsCH_3_SO_3_, 10 mM CsCl, 10 mM KCl, 1 mM MgCl_2_ and 5 mM HEPES, and the pH was adjusted to 7.35 using CsOH. Then, 100 µg/mL nystatin was added to the intracellular solution for perforation. Pulses depolarized from −70 to 0 mV by a voltage clamp were used to induce exocytosis. The stimulus train (Cm5+14) consisted of five 50 ms pulses followed by fourteen 500 ms pulses (100 ms intervals between pulses) to completely deplete the readily releasable pool (RRP) of vesicles.

### 2.6. Fluorescence Imaging of cAMP

Live-cell imaging of cAMP dynamics was performed using RAB-ICUE [28], a genetically encoded fluorescent cAMP indicator, in which amino acids 149–881 of Epac1 are sandwiched between a dimerization-dependent red fluorescent protein (ddRFP) pair [29]. cAMP binding to RAB-ICUE causes a conformational change that reduces ddRFP dimerization and thus decreases the fluorescence intensity. Single β-cells were transfected with RAB-ICUE using the 10-μL neon transfection system kit (MPK1096, Invitrogen, Waltham, MA, USA). After 48 h, the transfected β-cells were observed under a spinning disk confocal microscope (Olympus) equipped with a 40 × 1.35 numerical aperture (NA) oil objective lens. A 561-nm laser was used to excite RAB-ICUE fluorescence, and an Andor iXon3 897 EMCCD camera was used to collect emitted photons. The temperature of the extracellular solution (HBSS, 14025076, GIBCO, Grand Island, NE, USA) was maintained at 30–37 °C. Stimulation solutions (HBSS containing 100 nM glucagon with 1 µM MK0893 or 1 µM Exendin 9-39) and inhibition solutions (HBSS containing 100 µM NB001, 10 µM NKY80, or 10 µM KH7) were supplied to cells using a multichannel microperfusion system (MPS-2, Biogo, Wuhan, China). Because the fluorescence intensity of RAB-ICUE decreased when cAMP levels were elevated, we inverted the RAB-ICUE intensity curves to visualize the responses better. The key reagent information is as followed (Table 2).

### 2.7. RNA Extraction and Quantitative Real-Time PCR

Total RNA was extracted from isolated islets using the RNeasy Mini Kit (74104, QiAGEN, Hilden, Germany). First-strand complementary DNA was synthesized from total RNA using the TransScript One-Step gDNA Removal and cDNA Synthesis SuperMix (AT311-03, TransGenBiotech, Beijing, China). Real-time PCR was performed on an Eppendorf RealPlex2 system using TransStart Top Green qPCR supermix (AQ131-03, TransGenBiotech, Beijing, China). The qPCR primers used to detect *Gcgr* expression were *Gcgr*-F (5′-TTGCCACCTTCTCTGAGAGG-3′) and *Gcgr*-R (5′-GTAAGGCCAGGAAGACAGGA-3′). The qPCR primers used to detect *Glp1r* expression were *Glp1r*-F (5′-CCTGTCGGAGTGTGAAGAGT-3′) and *Glp1r*-R (5′-GCAAGTGTCTGAAGCCAACA -3′). RNA transcript levels were quantified using the 2^–ΔΔCt^ method by normalizing with GAPDH. Then, the results were normalized to the *Gcgr^βcell+/+^* control.

### 2.8. Pancreatic Sections and Immunohistochemistry

Pancreases isolated from 16-week-old male mice were weighed, fixed in 4% paraformaldehyde for 24 h, and then embedded in paraffin. Then, 5 µm-thick sections were collected at 100–150 µm intervals between two adjacent sections throughout the whole pancreas. Immunohistochemistry analysis was performed as previously described [30] using a mouse anti-insulin antibody (1:200, ZSGB-BIO, Beijing, China) and a rabbit anti-glucagon antibody simultaneously (1:200, ZSGB-BIO, Beijing, China). Finally, insulin-positive cells were colored red, while glucagon-positive cells were colored golden brown. Quantitative image analysis was performed using ImagePro Plus 6.0 software.

### 2.9. Statistics

All replicates are from, minimally, three separate experiments. All data were analyzed using SigmaPlot and GraphPad 7.0 software. The results are presented as the mean ± SEM. Statistical significance was evaluated using Student’s *t*-test for single Gaussian-distributed datasets or the Mann–Whitney rank-sum test for non-single Gaussian-distributed datasets. * *p* < 0.05, ** *p* < 0.01, *** *p* < 0.001.

## 3. Results

### 3.1. Glucagon Potentiates Insulin Secretion Via β-Cell GCGR at Physiological Concentrations of Glucose

**Figure 1:**
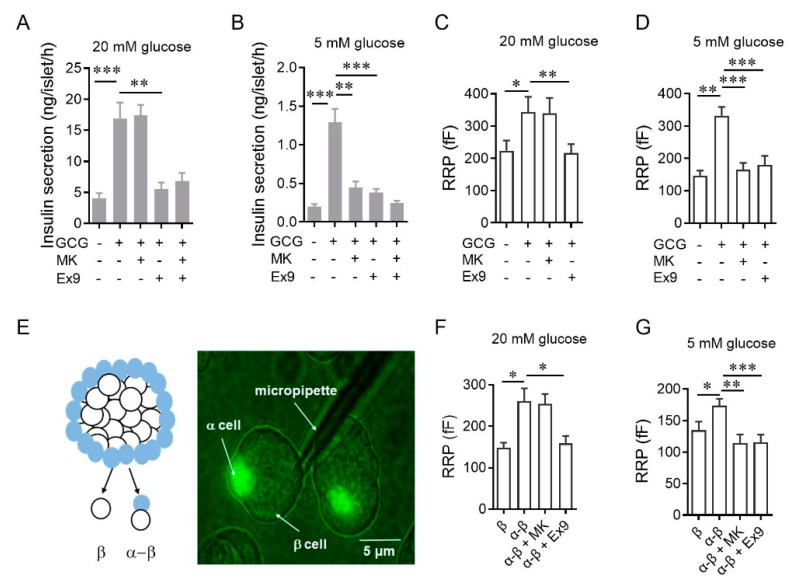
Glucagon potentiates insulin secretion via β-cell glucagon receptors (GCGR) at physiological doses of glucose. (**A**,**B**) Effects of MK0893 or Exendin 9-39 on 100 nM glucagon-induced insulin secretion of isolated mouse islets in 20 mM (**A**) and 5 mM glucose (**B**) (*n* = 5–7 per group). Glucagon is abbreviated as GCG, MK0893 is abbreviated as MK, and Exendin 9-39 is abbreviated as Ex9 in the figures. (**C**,**D**) Effects of MK0893 or Exendin 9-39 on 100 nM glucagon-increased RRP size of single β-cells isolated from C57BL/6J mice in 20 mM glucose (**C**) or 5 mM glucose (**D**) (*n* = 16–25 per condition). (**E**) Perforated patch on a α-β cell cluster. Left panel: schematic depicting the dissociation of GYY mouse islets into a α-β cell cluster or a single β-cell. Right panel: representative image of a perforated patch performing on a α-β cell cluster. (**F**,**G**) Effects of MK0893 and Exendin 9-39 on the RRP size of β-cells in α-β cell clusters isolated from GYY mice in 20 mM glucose (**F**) or 5 mM glucose (**G**) (*n* = 16–23 per condition). Statistical significance was evaluated using Student’s *t*-test for single Gaussian-distributed datasets or the Mann–Whitney rank-sum test for non-single Gaussian-distributed datasets. * *p* < 0.05, ** *p* < 0.01, *** *p* < 0.001.

To dissect the downstream receptor of glucagon, we measured insulin secretion from islets in the presence of either a GCGR antagonist (MK0893) or a GLP-1R antagonist (Exendin 9-39) (Table 1). At 20 mM glucose, glucagon-stimulated insulin secretion was abolished by Exendin 9-39 but not MK0893 (Figure 1A), consistent with the dispensable role of GCGR previously reported [18,19]. However, reducing the incubating glucose concentration to 5 mM made the glucagon effects susceptible to either GLP-1R or GCGR inhibition (Figure 1B). These results indicate that glucagon stimulates insulin secretion through GLP-1R at 20 mM glucose, but through GLP-1R and GCGR at 5 mM glucose. To exclude the possibility that the variable glucagon actions were due to the β-cells’ heterogeneity within an islet, we used membrane capacitance recording to measure the size of the readily releasable pool (RRP) of exocytotic vesicles in individual mouse β-cells [31] (Figure S1). Under the perforated patch-clamp configuration, cytosolic contents with the cell were largely intact, which allowed for modulation of RRP by glucose and glucagon to be quantified. Again, glucagon-enhanced RRP from single β-cells in the presence of 20 mM glucose was only sensitive to Exendin 9-39, while both MK0893 and Exendin 9-39 treatment eliminated the stimulatory effects of glucagon on RRP at 5 mM glucose (Figure 1C,D). Taken together, the results with the supraphysiological dose of glucagon indicate glucagon stimulates insulin secretion through GLP-1R and GCGR at 5 mM glucose, whereas 20 mM glucose overrides the effects of GCGR.

We then tried to investigate whether endogenous glucagon released from α-cells also modulates neighboring β-cell secretions through the same mechanisms. We used GluCre-ROSA26EYFP mice, in which α-cells were labeled with eYFP [32] (Figure 1E), and compared the secretory abilities of single β-cells with those of α-β clusters. The mean size of the RRP in β-cells within α-β clusters was significantly larger than that in single β-cells, which was abolished by Exendin 9-39 but not by MK0893 at 20 mM glucose (Figure 1F). This contrasts with the robust suppression of glucagon facilitated secretion by applying either MK0893 or Exendin 9-39 at 5 mM glucose (Figure 1G). Overall, these data confirm that glucagon is a dual-receptor agonist that consistently activates GLP-1R to promote insulin secretion in the 5–20 mM glucose range and enhances insulin secretion via β-cell GCGR at physiological glucose concentrations.

### 3.2. High Glucose Concentration Evokes cAMP Elevation via AC5 in β-Cells, Which Is Also Downstream of GCGR Activation, thus Bypassing GCGR in Promoting Insulin Secretion

**Figure 2:**
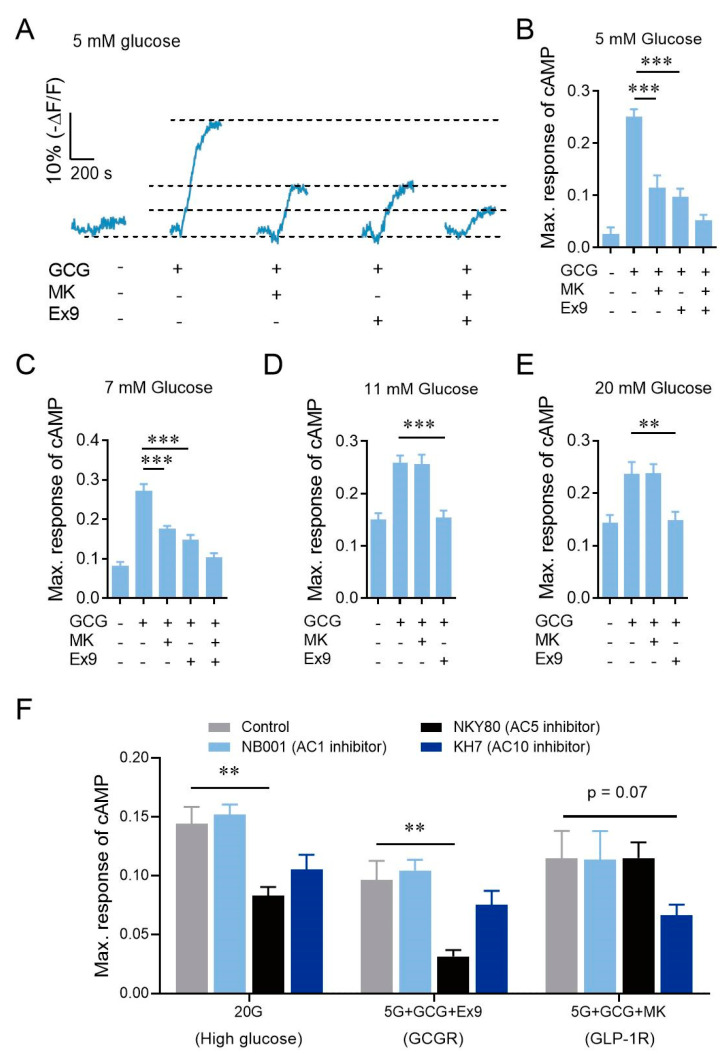
High glucose activates the same ACs as GCGR activation to increase the cAMP signal. (**A**) Average curves of intracellular cAMP signaling in β-cells induced by 100 nM glucagon and effects of MK0893 or Exendin 9-39 on glucagon-induced cAMP signaling in 5 mM glucose (*n* = 17–21 per condition). (**B**) Maximum responses of intracellular cAMP signaling calculated from (**A**). (**C**–**E**) Maximum responses of intracellular cAMP signaling in β-cells induced by 100 nM glucagon and effects of MK0893 or Exendin 9-39 on glucagon-induced cAMP signaling in 7 mM glucose (**C**), 11 mM glucose (**D**), 20 mM glucose (**E**) (*n* = 15–19 per condition). (**F**) Effects of different adenylate cyclase (AC) inhibitors on high glucose-, GCGR- and GLP-1R-stimulated cAMP elevations (*n* = 17–19 per condition). 100 μM NB001, 10 μM NKY80, and 10 μM KH7 inhibit AC1, AC5, and AC10, respectively. Statistical significance was evaluated using Student’s *t*-test for single Gaussian-distributed datasets or the Mann–Whitney rank-sum test for non-single Gaussian-distributed datasets. ** *p* < 0.01, *** *p* < 0.001.

The stimulatory effects of GCGR on β-cells diminished at 20 mM glucose stimulation was unexpected and puzzling (Figure 1A,C,F). To explore the possible mechanism, we transfected primary mouse β-cells with RAB-ICUE [28], a genetically encoded cAMP fluorescence indicator. Additional glucagon stimulated a pronounced elevation of cAMP signal that was almost abolished when MK0893 and Exendin 9-39 were used together at 5 mM glucose (Figure 2A,B). Application of either antagonist significantly reduced but did not block the glucagon-evoked cAMP signal elevation, demonstrating additive effects of GCGR and GLP-1R at 5 mM glucose (Figure 2A,B). On the other hand, β-cells stimulated with 7, 11, or 20 mM glucose alone exhibited elevated cytosolic cAMP signals, which were further potentiated by the application of glucagon (Figure 2C–E). Both GCGR and GLP-1R activation contributed to glucagon-stimulated cAMP elevation at 7 mM glucose, similar to that detected at 5 mM glucose (Figure 2C). However, at 11 and 20 mM glucose, GCGR inhibition failed to reduce high glucagon-stimulated cAMP elevation, which was eliminated by suppressing GLP-1R function (Figure 2D,E). We hypothesize that the reason that GCGR activation further increases cAMP at physiological but not at high glucose concentrations is that GCGR and high glucose may recruit the same AC subtype to transform ATP into cAMP. We used NB001, NKY80, and KH7 to inhibit AC1, AC5, and AC10, respectively (Table 2). NKY80 inhibited high glucose- or GCGR- but not GLP-1R-stimulated cAMP elevation (Figure 2F), indicating that AC5 is the common downstream target of GCGR activation and high glucose stimulation. Our results suggest that under high glucose stimulation, a high level of ATP occupied all AC5 to synthesize cAMP, and GCGR could not recruit more AC5 and thus could not further increase cAMP level.

### 3.3. The Physiological Function of β-Cell GCGR Becomes Upregulated and More Essential to Glucose Homeostasis in Mice Fed HFD

**Figure 3:**
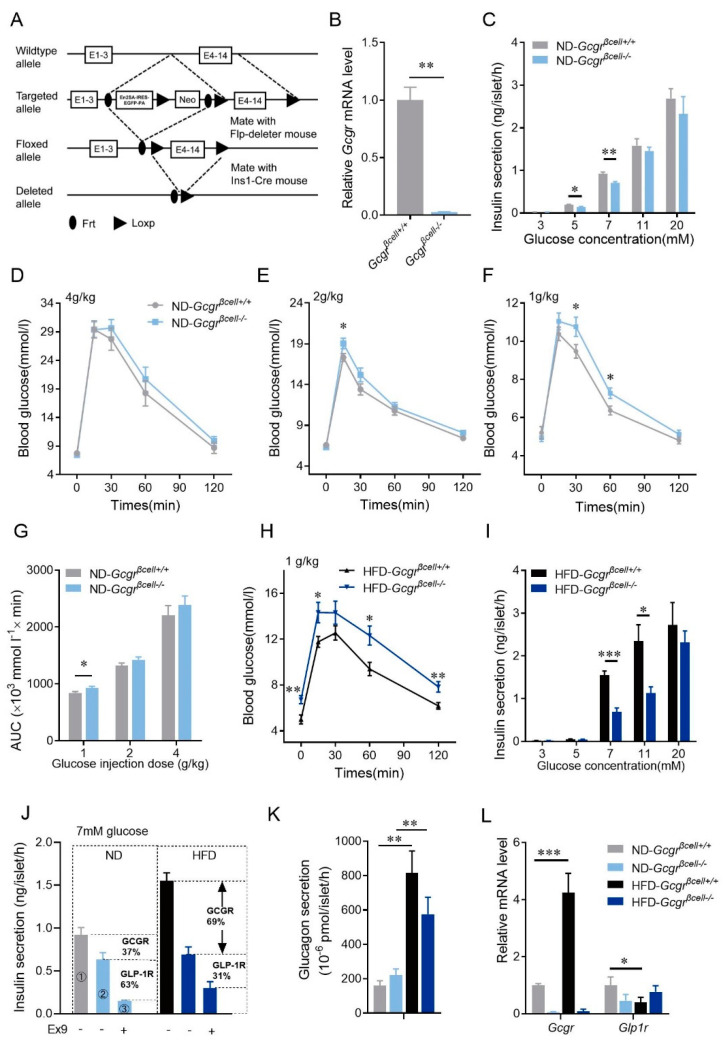
The physiological function of β-cell GCGR becomes upregulated and essential to glucose homeostasis in mice fed high-fat diets (HFD). (**A**) Schematic depicting the strategy for generating β-cell GCGR knockout mice. (**B**) Knockout efficiency of *Gcgr* mRNA in *Gcgr^βcell−/−^* mouse islets. (**C**) Insulin secretion of isolated islets from *Gcgr^βcell+/+^* and *Gcgr^βcell−/−^* mice fed the normal diet (ND) at different glucose concentrations (3 mM, 5 mM, 7 mM, 11 mM and 20 mM) (*n* = 5–9 per condition). (**D**–**F**) Intraperitoneal glucose tolerance tests (IPGTTs) on *Gcgr^βcell+/+^* and *Gcgr^βcell−/−^* mice fed the ND injected with different doses of glucose. The glucose doses were 4 g/kg (**D**), 2 g/kg (**E**), and 1 g/kg (**F**) (*n* = 12–14 mice per condition). (**G**) The areas under the curves (AUCs) of the intraperitoneal glucose tolerance tests (IPGTTs) on *Gcgr^βcell+/+^* and *Gcgr^βcell−/−^* mice fed the ND injected with different doses of glucose (1 g/kg, 2 g/kg, and 4 g/kg) (*n* = 12–14 mice per condition). (**H**) IPGTTs on *Gcgr^βcell+/+^* and *Gcgr^βcell−/−^* mice fed the HFD injected with 1 g/kg glucose (*n* = 12–15 mice per condition). (**I**) Insulin secretion of isolated islets from *Gcgr^βcell+/+^* and *Gcgr^βcell−/−^* mice fed the HFD at different glucose concentrations (3 mM, 5 mM, 7 mM, 11 mM and 20 mM) (*n* = 5–9 per condition). (**J**) Contributions of GCGR and GLP-1R on insulin secretion of isolated islets from *Gcgr^βcell+/+^* and *Gcgr^βcell−/−^* mice fed the ND or HFD in 7 mM glucose (*n* = 6–7 per condition). The proportions of GCGR and GLP-1R contributions are calculated as follows. The enhanced insulin secretion by glucagon is via activation of both GCGR and GLP-1R. Therefore, in mice fed the ND, the total amount of enhanced insulin secretion by glucagon equals the difference of insulin secretion between *Gcgr^βcell+/+^* islets and *Gcgr^βcell−/−^* islets pretreated with Exendin 9-39 (①−③). In this regard, the contribution of GCGR and GLP-1R were 37% ((①−②)/(①−③)) and 63% ((②−③)/(①−③)), respectively. The same method was applied on mice fed the HFD. (**K**) Glucagon secretion from isolated islets of *Gcgr^βcell+/+^* and *Gcgr^βcell-/-^* mice fed the ND or HFD under stimulation with 7 mM glucose (*n* = 4 per condition). (**L**) Relative mRNA levels of *Gcgr, Glp1r* in islets from *Gcgr^βcell+/+^* and *Gcgr^βcell−/−^* mice fed the ND or HFD (*n* = 3–4 per condition). Statistical significance was evaluated using Student’s *t*-test for single Gaussian-distributed datasets or the Mann–Whitney rank-sum test for non-single Gaussian-distributed datasets. * *p* < 0.05, ** *p* < 0.01, *** *p* < 0.001.

As high glucose bypassed the action of β-cells’ GCGR on cAMP elevation in vitro, we further examine whether β-cells’ GCGR physiologically modulate glucose homeostasis in vivo. We created β-cell-specific GCGR knockout mice (*Gcgr^βcell−/−^*, genotype: Ins1-cre; *Gcgr^f/f^*) (Figure 3A,B), in which β-cell mass and islet architecture were similar to those of control mice (Figure S2A–E). 

Similar to the pharmacological experiments (Figure 1 and Figure 2B–E), isolated islets from β-cell-specific GCGR knockout mice showed compromised insulin secretion under 5- or 7-mM glucose stimulation, but similar GSIS to the control islets under 11 or 20 mM glucose (Figure 3C). Interestingly, although displayed normal glucose tolerance at regular doses of glucose intraperitoneal injection (4 g/kg, 2 g/kg, Figure 3D,E,G), *Gcgr^βcell−/−^* mice became glucose-intolerant after injection of a small dose of glucose (1 g/kg, Figure 3F,G). Consistent with the data of isolated islets and β-cells, this result reinforced the conclusion that endogenously released glucagon enhances β-cell secretion and regulates blood glucose via the GCGR pathway at physiological glucose levels. 

In contrast to the subtle phenotype under the ND, *Gcgr^βcell−/−^* mice fed HFD had more severe hyperglycemia and glucose intolerance (Figure 3H and Figure S3A–C). Along with this phenotype, insulin secretion evoked by 7 mM and 11 mM glucose was reduced by half in the islets of *Gcgr^βcell−/−^* mice fed HFD compared to the control mice fed HFD (Figure 3I), and the differences were much higher than those between *Gcgr^βcell−/−^* and control mice fed ND (Figure 3C,I). By comparing the insulin secretion evoked by 7 mM glucose or 7 mM glucose combined with Exendin 9-39 from *Gcgr^βcell+/+^* and *Gcgr^βcell−/−^* islets, we concluded that GCGR and GLP-1R contributed 37% and 63% of the stimulatory effects of glucagon in mice fed ND, respectively. In mice fed HFD, these numbers changed to 69% and 31%, respectively (Figure 3J), highlighting the critical up-regulation of β-cell GCGR function in maintaining glucose tolerance under HFD. In line with these data, compared to the controls fed ND, we also observed a 3–4-fold increase in glucagon released from islets isolated from HFD mice (Figure 3K), significant upregulation of *Gcgr* mRNA, and downregulation of *Glp-1r* mRNA in islets (Figure 3L).

Overall, and differently from previous studies [18,19,20,33], we have shown a powerful effect of glucagon-activated GCGR in potentiating insulin secretion at physiological glucose concentrations, possibly via the AC5 not used by the GLP-1R. Moreover, we demonstrate that the essential role of β-cells’ GCGR in regulating physiological glucose homeostasis becomes more critical during nutrient overload.

## 4. Discussion

The physiological function of GCGR on β-cells has not been elucidated, albeit glucagon stimulates insulin secretion. The effect of glucagon or α-cells on β-cell insulin secretion is thought to depend on GLP-1R. Here, our results highlight the vital role of GCGR on β-cells in regulating insulin secretion at the physiological condition and during metabolic stress.

Although whole-body GCGR knockout (*Gcgr*^−/−^) mice exhibited reduced GSIS [34], the phenotype was primarily due to alterations in islet structures induced by the absence of the liver GCGR [35,36]. GCGR on β-cells was believed dispensable for insulin secretion [18,19,20]. Our results here indicate that this dogma is only partially true. We suggest that the effect of GCGR activation on cAMP elevation is only dispensable at high glucose, but necessary at physiologically relevant doses of glucose. Most of the previous studies focused on insulin secretion stimulated by high glucose but neglected the effect of GCGR on insulin secretion stimulated by the physiological dose of glucose. The latter is difficult to be identified as, even in our study, *Gcgr^βcell−/−^* mice did not show glucose intolerance when, being IP, injected with 2 g/kg and 4 g/kg glucose. The phenotype of glucose intolerance was only identified when injected with 1 g/kg glucose. We conducted some preliminary exploration of the underlying mechanisms, finding that 20 mM glucose and GCGR mobilized the same pool of ACs, AC5 (Figure 2F). High glucose metabolism produces a lot of ATP, thus the limiting factor is the number of recruited ACs that convert ATP into cAMP [37,38]. When high glucose activates all available AC5, GCGR does not further increase active AC5 to promote cAMP levels, and thus becomes dispensable at high glucose-stimulated insulin secretion. GLP-1R probably activates other ACs, such as AC10, further raising cAMP levels and thus enhancing high glucose-stimulated insulin secretion. At lower glucose conditions, due to the low ATP level, the number of active ACs and ATP level are the limiting factors of cAMP level. GCGR activation helps to recruit more AC5, thereby increasing cAMP levels and insulin secretion. Further studies are needed to confirm this hypothesis. 

In combination with *Gcgr^βcell−/−^* islets and Exendin 9-39, we found that the contribution ratio of GCGR and GLP-1R to glucagon-enhanced 7 mM glucose-stimulated insulin secretion was 37:63 in ND-fed mice (Figure 3J). Interestingly, the ratio was reversed to 69:31 in mice fed HFD (Figure 3J). This means that GCGR becomes more critical in amplifying GSIS under metabolic stress. This was consistent with the enhanced glucagon secretion, increased *Gcgr* gene expression and reduced *Glp1r* expression in the islets of mice fed HFD (Figure 3K,L). The underlying mechanisms need more studies to unravel. 

Glucagon acts as the natural dual-receptor agonist of both GLP-1R and GCGR. Designing drugs with the optimal combination of GLP-1R and GCGR activation is important and may confer greater benefits in reversing diabetes than GLP-1R activation alone, as demonstrated recently [39,40,41,42]. However, the more potent activation of GCGR by excessive glucagon may upset this intricate balance and lead to overall adverse and diabetogenic effects, such as inducing more glucose production in the liver, which participates in and contributes to the progression of T2D, as confirmed [43]. Our study demonstrates that the contribution of GCGR to insulin secretion is significantly increased relative to GLP-1R in HFD-fed mice. As human islets have more intermixed α-β cell interfaces [44,45,46], the proportion of GCGR:GLP-1R effect may be more critical for human islet function to keep a narrower normal glycemic fluctuation. Therefore, we suggest that the changes in the effects of GCGR and GLP-1R induced by metabolic stress should also be considered when designing GCGR- and GLP-1R-combined agonists for the treatment of diabetes. 

## Figures and Tables

**Table 1:** The information of glucagon-related drugs.

Reagent	Source	Identifier
glucagon	Sigma	G2044
MK0893	Medchem Express	HY-50663
Exendin 9-39	Sigma	E7269

**Table 2:** The information of adenylyl cyclase (AC) subtype inhibitors.

Reagent	Source	Identifier
NB001	MedChemExpress	HY-14425
NKY80	Cayman	17,777
KH7	Cayman	13,243

## Data Availability

The data presented in this study are available on request from the corresponding author.

## Supplementary Materials

The following are available online at https://www.mdpi.com/article/10.3390/cells10092495/s1, Figure S1. Exocytosis signals of a representative β-cell induced by a stimulus protocol of pulses depolarized (−70 mV to 0 mV) via a voltage clamp in the perforated whole-cell configuration, Figure S2. The islet structure, number, and size in *Gcgr^βcell−/−^* mice, Figure S3. The AUCs, FPG and 2h-PG of *Gcgr^βcell−/−^* mice fed the ND or HFD.
