# Supplementary material for: Glucagon Potentiates Insulin Secretion Via β-Cell GCGR at Physiological Concentrations of Glucose
Source: Cells. 2021 Sep 21;10(9):2495. doi: 10.3390/cells10092495 (PMC8471175; doi:10.3390/cells10092495)
Supplement: Supplementary file 1 [file cells-10-02495-s001.zip › cells-1384918-supplementary.pdf]

Supplementary Materials

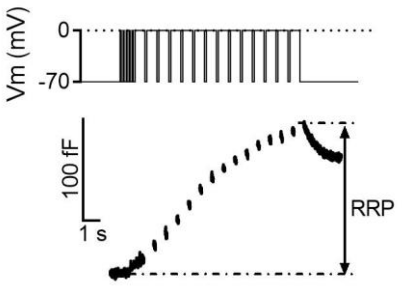

Figure S1. Exocytosis signals of a representative β-cell induced by a stimulus protocol of pulses depolarized (−70 mV to 0 mV) via a voltage clamp in perforated whole-cell configuration.
The stimulus train consisted of five 50-ms pulses followed by fourteen 500-ms pulses (100 ms intervals between pulses) to trigger vesicle secretion to assess the initial size of the RRP. The measurement of RRP was used as an assay of the individual β-cell secretory ability.

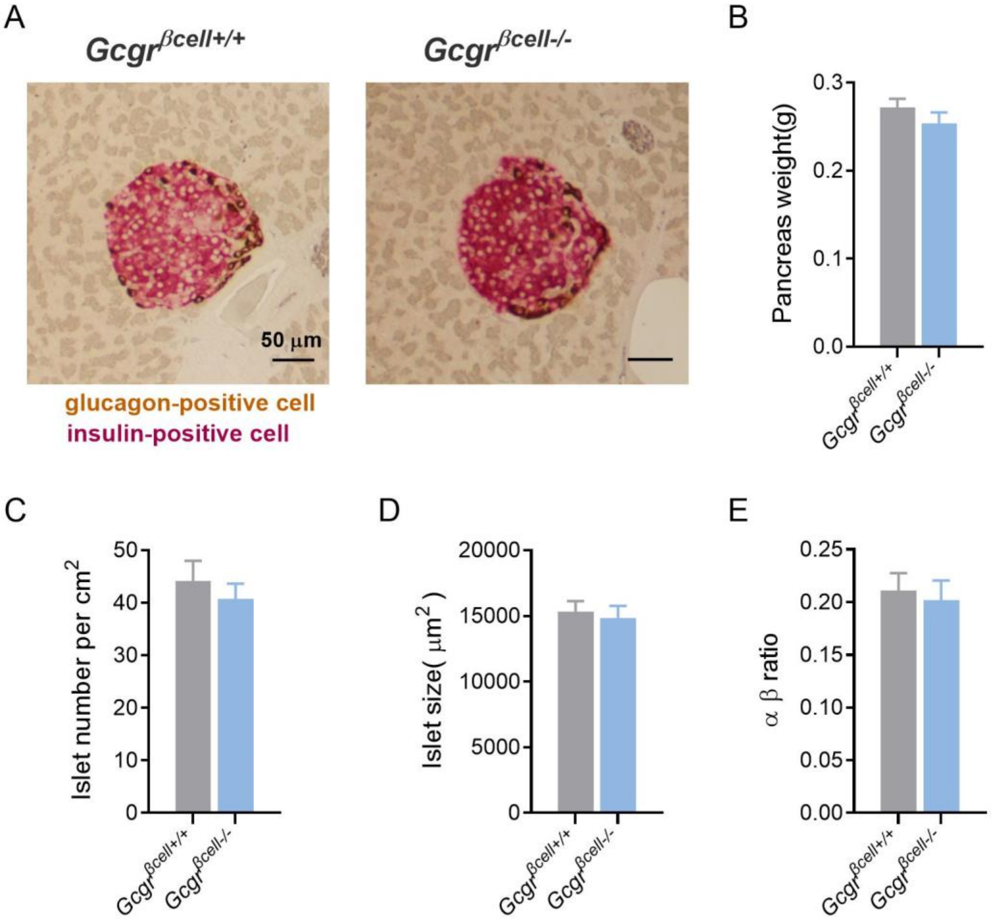

Figure S2. The islet structure, number, and size in *Gcgr*$^{\beta cell-/-}$ mice.
(**A**) Representative histological pancreatic sections from *Gcgr*$^{\beta cell+/+}$ and *Gcgr*$^{\beta cell-/-}$ mice double-stained with anti-glucagon (brown) and anti-insulin (red) antibodies (n=5 per group).
(**B-E**) Pancreas weights (**B**), islet numbers (**C**), islet sizes (**D**) and the α- to β-cell area ratios (**E**) in *Gcgr*$^{\beta cell+/+}$ and *Gcgr*$^{\beta cell-/-}$ mice (n=5 per group).

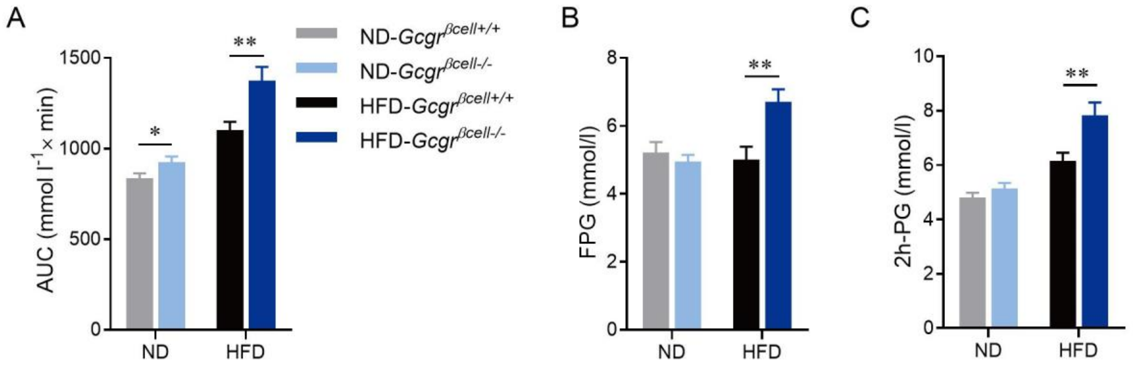

Figure S3. The AUCs, FPG and 2h-PG of *Gcgr*$^{\beta cell-/-}$ mice fed the ND or HFD.

(**A**) The areas under the curves (AUCs) in the IPGTTs with 1g/kg glucose in Figure 3F and 3H.

(**B**) The fasting plasma glucose (FPG) in the IPGTTs with 1g/kg glucose in Figure 3F and 3H.

(**C**) The 2 hour-plasma glucose (2h-PG) in the IPGTTs with 1g/kg glucose in Figure 3F and 3H.
